# Potential diagnostic value of pleural fluid cytokines levels for tuberculous pleural effusion

**DOI:** 10.1038/s41598-020-79685-1

**Published:** 2021-01-12

**Authors:** Neda Dalil Roofchayee, Majid Marjani, Neda K. Dezfuli, Payam Tabarsi, Afshin Moniri, Mohammad Varahram, Ian M. Adcock, Esmaeil Mortaz

**Affiliations:** 1 Department of Immunology, Faculty of Medicine, Shahid Beheshti University of Medical Sciences, Tehran, Iran; 2 Clinical Tuberculosis and Epidemiology Research Center, National Research Institute of Tuberculosis and Lung Diseases, Shahid Beheshti University of Medical Sciences, Tehran, Iran; 3 Mycobacteriology Research Center, National Research Institute of Tuberculosis and Lung Diseases (NRITLD), Shahid Beheshti University of Medical Sciences, Tehran, Iran; 4 National Heart and Lung Institute, Imperial College, London, UK; 5 Priority Research Centre for Asthma and Respiratory Disease, Hunter Medical Research Institute, University of Newcastle, Newcastle, NSW Australia; 6 Department of Immunology, School of Medicine, Dezful University of Medical Sciences, Dezful, Iran

**Keywords:** Immunology, Biomarkers, Diseases, Medical research

## Abstract

Patients with tuberculous pleural effusion (TPE) or malignant pleural effusions (MPE) frequently have similar pleural fluid profiles. New biomarkers for the differential diagnosis of TPE are required. We determined whether cytokine profiles in the PE of patients could aid the differential diagnosis of TPE. 30 patients with TPE, 30 patients with MPE, 14 patients with empyema (EMP) and 14 patients with parapneumonic effusion (PPE) were enrolled between Dec 2018 and 2019. The levels of interleukin (IL)-6, IL-18, IL-27, CXCL8, CCL-1 and IP-10 were determined in PE by ELISA along with measurements of adenosine deaminase (ADA). The best predictors of TPE were combined ADA.IL-27 [optimal cut-off value = 42.68 (10^3^ U ng/l^2^), sensitivity 100%, specificity 98.28%], ADA [cut off value 27.5 (IU/l), sensitivity 90%, specificity 96.5%] and IL-27 [cut-off value = 2363 (pg/ml), sensitivity 96.7%, specificity 98.3%, p ≤ 0.0001]. A high level of IL-6 [cut-off value = 3260 (pg/ml), sensitivity 100%, specificity 67.2%], CXCL8 [cut-off value = 144.5 (pg/ml), sensitivity 93.3%, specificity 58.6%], CCL1 [cut-off value = 54 (pg/ml), sensitivity 100%, specificity 70.7%] and IP-10 [cut-off value = 891.9 (pg/ml), sensitivity 83.3%, specificity 48.3%] were also predictive of TPE. High ADA.IL-27, ADA and IL-27 levels differentiate between TPE and non-TPE with improved specificity and diagnostic accuracy and may be useful clinically.

## Introduction

*Mycobacterium tuberculosis* (*Mtb*) is one of the oldest and most important human pathogens and infection with *Mtb* has a high global mortality rate^1^. Nearly one-third of the world's population is asymptomatically (latently) infected with tuberculosis, and about 3–10% of these people progress to active disease throughout their life. In 2018, 10 million people became infected with tuberculosis and 1.5 million died including 0.3 million people co-infected with HIV (https://www.who.int/tb/global-report-2019).

Tuberculosis has two forms in humans based on the affected organs: pulmonary and extra-pulmonary tuberculosis. In the extra-pulmonary form, many organs may be involved but importantly pleural effusions (PE) are an important manifestation of the disease^2^. In addition to TB, malignancy, cardiovascular disease and infections can also result in PE. Various methods including biochemical tests, cytology, bacterial culture and biopsy examination are used to determine the cause of PE. However, in many cases the etiology remains ill-defined^2,3^. Differential diagnosis of tuberculous PE (TPE) from other PE, especially malignant pleural effusions (MPE), is challenging clinically^3^ as both TPE and MPE are lymphocytic in origin^2^. The gold standard for differentiating TPE from other pleural effusions with different etiologies is the isolation *Mtb* from either pleural fluid or pleural biopsy (100% specificity)^4^. Although culturing of sputum has a diagnostic value with 100% specificity, it is time consuming and delays the diagnosis. Manifestations of granuloma (~ 95%), provided that other causes of granulomatosis are discounted, is also used to diagnose TPE but is considered as an invasive approach^5^.

TPE is a delayed hypersensivity reaction to *Mtb* and the result of a pathological immune response associated with increased cytokines including interleukins (ILs) and chemokines^6–8^. Based on previous studies^9–13^ we hypothesized that cytokine and chemokine levels in TPE may differentiate this disease from other causes of PE. Therefore, the objective of this study was to measure the levels of adenosine deaminase (ADA), cytokines and chemokines (IL-6, IL-18, IL-27, CXCL8, CCL1 and IP-10) in the pleural fluid as a relatively rapid laboratory method for differentiating between TPE and non-TPE. In the current study, we shown that levels of ADA, IL-27 and combined ADA.IL-27 have the best sensitivity, specificity and predictive value to diagnosis of TPE.

## Results

**Figure 1:**
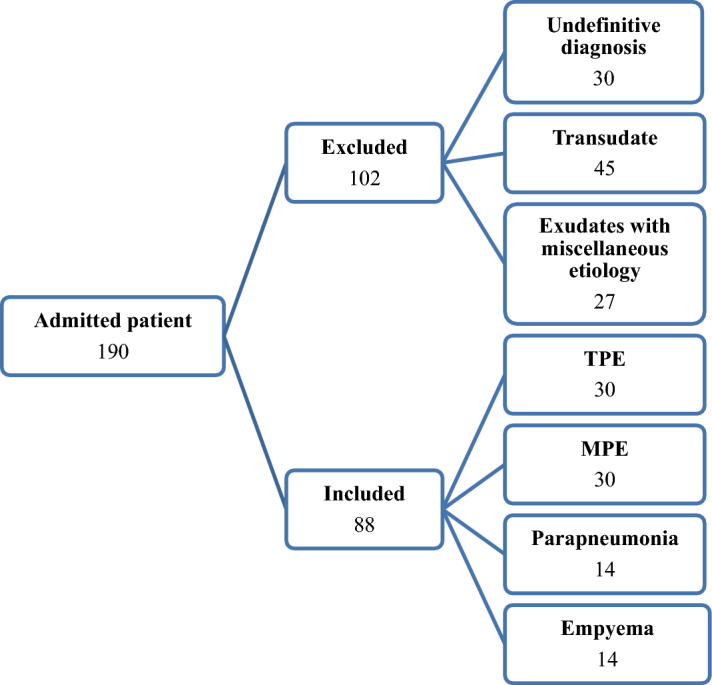
Flow chart of study groups.

190 patients with unknown PEs were enrolled in this study. After diagnosing the etiology of the PEs we excluded 102 subjects who failed to meet the diagnostic criteria (30), provided transudate effusions (45) or had exudates with miscellaneous etiology (27). The number of PEs with miscellaneous etiologies in each group was not suitable for statistical analysis (Fig. 1). The remaining 88 patients with exudate PEs were classified into 4 diagnostic groups: TPE, MPE, empyema (EMP) and parapneumonic effusion (PPE). Thirty HIV negative patients, aged 18–84 years, with a positive *Mtb* test in biopsy specimens and pleural tissue granuloma were included in the TPE group. 30 patients, aged 32–80 years, newly diagnosed with MPE based on histologically analysis: 15 cases had adenocarcinoma, 10 patients had squamous cell carcinoma (SCC) and 5 patients suffered from non-squamous cell carcinoma (NSCC). 14 patients with EMP, aged 20–75 years, and 14 patients with PPE were included. The demographic data of the patients and their biochemical characteristics are given in Tables 1 and 2. The distribution of the cytokines and chemokines in each group of subjects are summarized in Table 3.

**Table 1:** Demographic data of the study population.

	Patients	TPE	MPE	EMP	PPE	p value†
N	88	30	30	14	14	
Age, yr	56.6 ± 1.9	52.3 ± 3.9	59.5 ± 2.8	56.0 ± 4.2	59.9 ± 3.2	0.3676
Sex (F/M), n	43/45	13/17	19/11	6/8	5/9	

Values are presented as mean ± standard error of the mean (SEM).

*TPE* tuberculous pleural effusion, *MPE* malignant pleural effusion, *EMP* Empyema, *PPE* parapneumonic.

^†^Comparisons of data between TPE, MPE, EMP and PPE effusions were performed using one-way analysis of variance.

**Table 2:** Biochemical and cytological characteristics of pleural effusions*.

	TPE	Non-TPE	p value*	
LDH (IU/l)	631.9 ± 19.7*	456.4 ± 17.5	≤ 0.0001	
Protein (g/l)	39.2 ± 1.78	33.40 ± 1.14	0.0055	

	TPE	MPE	EMP	PPE
LDH (IU/l)	631.9 ± 19.7^†^	382.8 ± 10.6	657.4 ± 18.2	413.2 ± 24.4
Protein (g/l)	39.2 ± 1.78^†^	33.0 ± 1.5	35.9 ± 2.3	31.8 ± 2.6
**Differential cell counts, %**				
Lymphocytes cells	72.8 ± 1.07^†^	53.5 ± 0.7	14.4 ± 0.8^†^	19.3 ± 0.9^†^
Neutrophils cells	8 ± 0.4	5.9 ± 0.3	78.4 ± 0.9^†^	53.7 ± 1.1
Macrophage cells	12.5 ± 0.4^†^	31.2 ± 0.4^†^	5.7 ± 0.5^†^	8.6 ± 0.5
Mesothelial cells	3.1 ± 0.2^†^	6.6 ± 0.3^†^	5.7 ± 0.3^†^	1.6 ± 0.3^†^
Malignant cells	–	3.5 ± 0.3	–	–

*Values are presented as mean ± standard error of the mean (SEM). Comparisons of data between TPE and non-TPE groups were performed using Student’s *t* test.

^†^Values are shown as mean ± SEM.

*TPE* tuberculous pleural effusion, *MPE* malignant pleural effusion, *EMP* Empyema, *PPE* Parapneumonic.

**Table 3:** The concentrations of the ADA, cytokines and chemokines in pleural effusions*.

	TPE	MPE	p value	EMP	p value	PPE	p value	Non-TPE	p value
IL-27 (pg/ml)	4725 (3993–7598)	838.5 (715.5–967.5)	≤ 0.0001	1403 (1163–1720)	≤ 0.0001	1409 (1003–1699)	0.002	978 (835.3–1401)	≤ 0.0001
IL-6 (pg/ml)	5735 (4935–6302)	1240 (783.3–1955)	≤ 0.0001	4114 (3413–5150)	≤ 0.0001	2875 (1957–4167)	≤ 0.0001	2273 (1121–3832)	≤ 0.0001
IL-18 (pg/ml)	2196 (513.4–3035)	568.5 (395–800.3)	0.0008	2409 (1156–4595)	0.2522	2196 (1487–2663)	0.5539	1003 (577.8–2291)	0.1797
CXCL8 (pg/ml)	1038 (344.1–1853)	125 (66.5–599.6)	≤ 0.0001	184.5 (114.1–1041)	0.0093	98.02 (50.25–326.5)	≤ 0.0001	123 (67.75–485.6)	≤ 0.0001
CCL1 (pg/ml)	565 (104.8–2353)	12.21 (6.4–23.35)	≤ 0.0001	42.9 (30.18–61.98)	0.0195	126.3 (42.65–392.3)	≤ 0.0001	32.2 (12.2–61.98)	≤ 0.0001
IP-10 (pg/ml)	2833 (909–4184)	662 (300.7–1524)	≤ 0.0001	1503 (1157–3323)	0.3892	823.4 (484.9–2286)	0.0101	944.4 (355.5–1970)	0.0001
ADA (IU/l)	42.73 ± 1.71	14.5 ± 0.6	≤ 0.0001	25 ± 1.11	≤ 0.0001	21.21 ± 0.32	≤ 0.0001	18.9 ± 0.07	≤ 0.0001
ADA.IL-27 (10^3^ ng IU/l^2^)	208.5 (178.2–548.8)	12.2 (9.15–13.53)	≤ 0.0001	33.86 (26.46–4.52)	≤ 0.0001	30.25 (21.57–35.23)	≤ 0.0001	19.23 (13.33–48.96)	≤ 0.0001

All values except those for ADA and ADA.IL-27 are presented as the median and 25–75% percentile and comparisons made between TPE and the other groups were performed using the Mann–Whitney U test.

Values for ADA and ADA.IL-27 are shown as mean ± SEM. Comparisons of data between TPE and non-TPE groups were performed using the Student’s *t* test.

*IL* interleukin, *CXCL* C-X-C motif chemokine ligand, *CCL* C–C motif chemokine ligand, *IP-10* interferon gamma-induced protein 10 or CXCL10, *ADA* adenosine deaminase.

### ADA levels can discriminate between TPE and MPE

**Figure 2:**
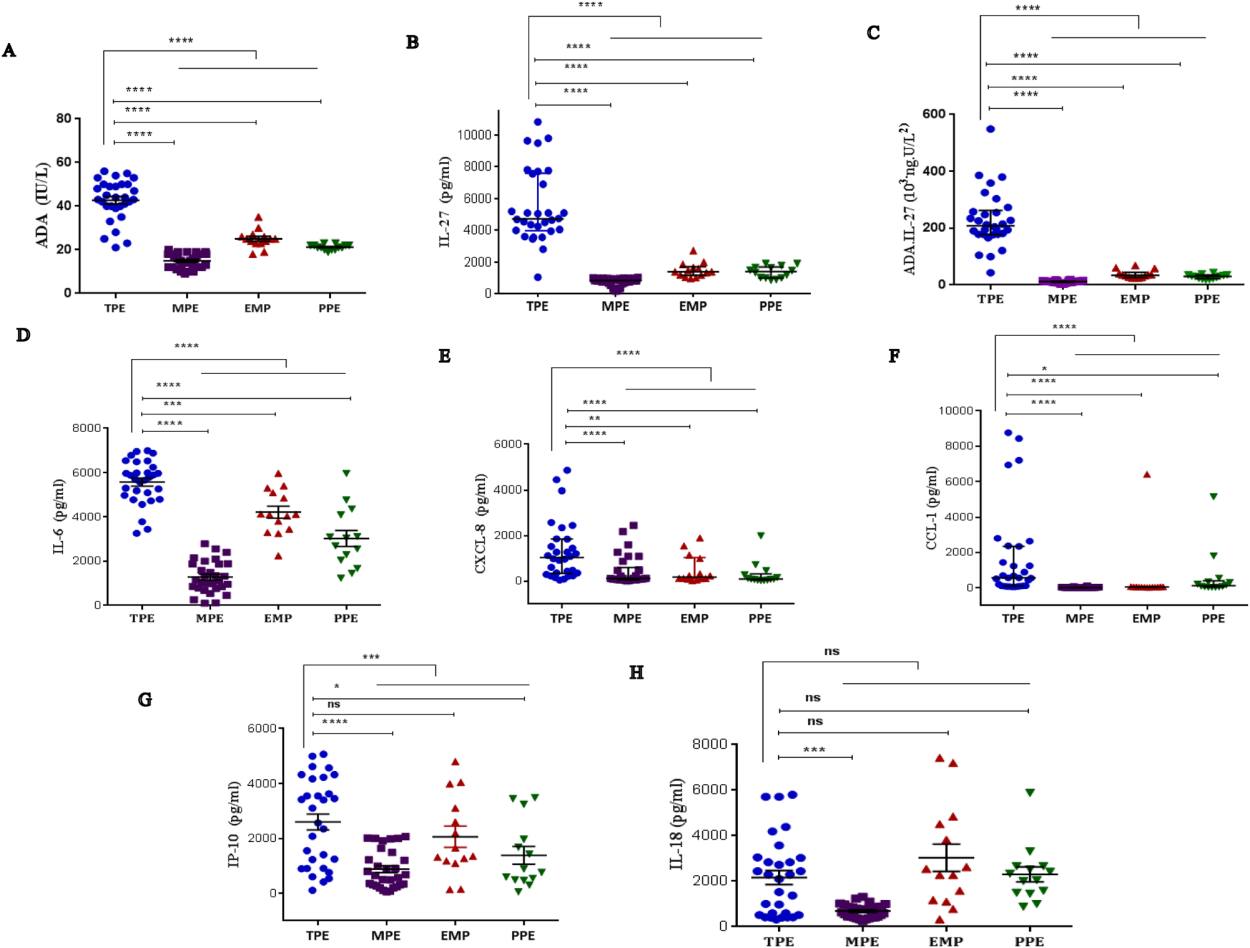
(**A**) ADA pleural fluid concentrations in TPE (n = 30) and in 3 etiologies of pleural effusions: malignant (n = 30), empyema (n = 14) and parapneumonic (n = 14) pleural effusions. In each data bar, Horizontal bars indicate mean and the bottom and top of the bar represent the standard error of the mean (SEM). (**B**) IL-27 pleural fluid concentrations in tuberculous pleural effusion (n = 30) and in 3 etiologies of pleural effusions: malignant (n = 30), empyema (n = 14) and parapneumonic (n = 14) pleural effusions. (**C**) ADA.IL-27 pleural fluid concentrations in tuberculous pleural effusion (n = 30) and in 3 etiologies of pleural effusions: malignant (n = 30), empyema (n = 14) and parapneumonic (n = 14) pleural effusions. (**D**) IL-6 pleural fluid concentrations in TPE (n = 30) and in 3 etiologies of pleural effusions: malignant, empyema and parapneumonic pleural effusions. (**E**) CXCL8 pleural fluid concentrations in TPE (n = 30) and in 3 etiologies of pleural effusions: malignant (n = 30), empyema (n = 14) and parapneumonic (n = 14) pleural effusions. (**F**) CCL1 pleural fluid concentrations in TPE (n = 30) and in 3 etiologies of pleural effusions: malignant (n = 30), empyema (n = 14) and parapneumonic (n = 14) pleural effusions. (**G**) IP-10 pleural fluid concentrations in TPE (n = 30) and in 3 etiologies of pleural effusions: malignant (n = 30), empyema (n = 14) and parapneumonic (n = 14) pleural effusions. (**H**) IL-18 pleural fluid concentrations in TPE (n = 30) and in 3 etiologies of pleural effusions: malignant (n = 30), empyema (n = 14) and parapneumonic (n = 14) pleural effusions.

**Figure 3:**
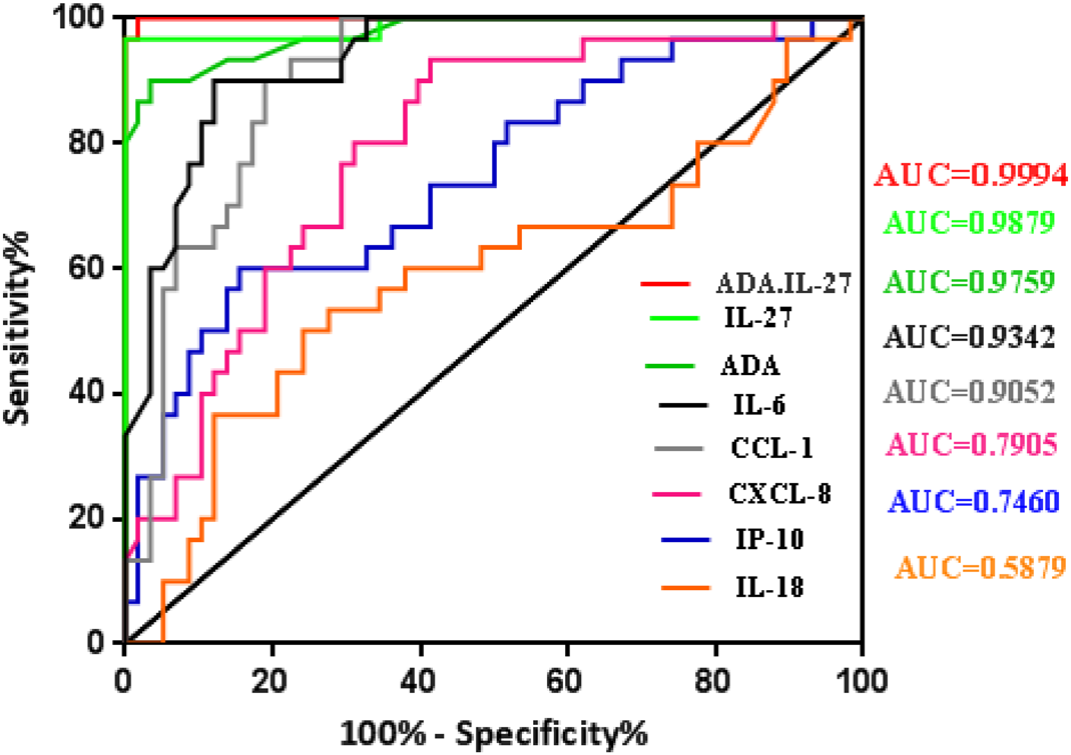
ROC curve of ADA, IL-27, ADA-IL-27, IL-6, IL-18, CXCL8, CCL1 and IP-10 for differential diagnosis of TPE (n = 30) versus non-TPE (n = 58).

Patients with TPE show a significant elevation of pleural protein (p = 0.0055) and LDH (p ≤ 0.0001) (Table 2). The levels of adenosine deaminase (ADA) in TPE (42.73 ± 1.71 IU/l) were also significantly higher than in non-TPE subjects [18.86 ± 0.7045 (IU/L), p ≤ 0.0001] (Table 2, Fig. 2A). The area under curve (AUC) for ADA to differentiate TPE from non-TPE was 0.975 (95% confidence interval 0.9471–1.005; p ≤ 0.0001) (Fig. 3). With a cut-off value of 27.5 (IU/l), we obtained a sensitivity of 90%, a specificity of 96.5%, together with positive likelihood ratios (PLR = 26.1), a negative likelihood ratio (NLR = 0.1), a positive predictive value (PPV = 93.1), a negative predictive value (NPV = 94.9) and a diagnostic accuracy of 94.3% (Table 4).

**Table 4:** The diagnostic accuracy of IL-6, IL-18, IL-27, CXCL8, CCL1, IP-10 and ADA in the differentiation of tuberculous from non-tuberculous effusions (Malignant, empyema and parapneumonic effusions).

Variables	Cut-off value	Area under curve(95% confidence interval)	p value	Sensitivity (%)	Specificity (%)	Positive likelihood ratio	Negative likelihood ratio	Positive predictive value	Negative predictive value	Diagnostic accuracy (%)
**TPE and non-TPE**										
IL-27	> 2363 pg/ml	0.9879	≤ 0.0001	96.67	98.28	56.07	0.03	96.6	98.2	97.72
IL-6	> 3260 pg/ml	0.9342	≤ 0.0001	100	67.24	3.03	0.0	61.2	100	74.4
IL-18	> 2285 pg/ml	0.5879	0.1797	50	75.9	2.07	0.66	51.7	74.5	67.04
CXCL8	> 144.5 pg/ml	0.7905	≤ 0.0001	93.3	58.6	2.256	0.11	53.8	94.4	70.45
CCL1	> 54 pg/ml	0.905	≤ 0.0001	100	70.69	3.41	0.0	63.8	100	80.68
IP-10	> 891.9 pg/ml	0.746	0.0001	83.33	48.28	1.61	0.35	45.5	84.8	60.2
ADA	> 27.5 IU/l	0.9759	≤ 0.0001	90	96.5	26.1	0.1	93.1	94.9	94.3
ADA.IL-27	> 42.68 10^3^ U ng/l^2^	0.9994	≤ 0.0001	100	98.28	58.13	0.0	96.78	100	98.86

*ADA* adenosine deaminase, *IL* interleukin, *CXCL* C-X-C motif chemokine ligand, *CCL* C–C motif chemokine ligand, *IP-10* interferon gamma-induced protein 10 or CXCL10.

### Cytokine levels in tuberculous and non-tuberculous pleural fluid

IL-27, combined ADA.IL-27, IL-6, CXCL8, CCL1 and IP-10 in the TPE and non-TPE (MPE, EMP, PPE) groups were all detected in the pleural fluid. The concentrations of all these cytokines were significantly higher in the TPE group than in the non-TPE groups (Table 3). In contrast, the concentration of IL-18 in the TPE group was not significantly different from non-TPE groups (Table 3).

The median IL-27 concentration in the TPE group was 4725 (pg/ml) [25–75% percentile, 3993–7598 (pg/ml)] was significantly higher than in non-TPE groups [978 (pg/ml), 25–75% percentile, 835.3–1401 (pg/ml); p ≤ 0.0001] (Table 3, Fig. 2B). This gave a high AUC value of 0.9879 (95% CI 0.9640–1.012, p ≤ 0.0001) (Fig. 3). Furthermore, we determined the optimal IL-27 cut-off value in the pleural fluid as 2363 (pg/ml) by ROC curve. With this cut-off value, a sensitivity of 96.67% (95% CI 82.78–99.92%), a specificity of 98.28% (95% CI 90.76–99.96%), together with a PLR = 56.07, a NLR = 0.03, a PPV = 96.6 and a NPV = 98.2 for TPE diagnosis was obtained compared with non-TPEs. Thus, the diagnostic accuracy of IL-27 levels in pleural effusion was 97.72% (86/88) (Table 4).

The median IL-6 level in the TPE group was 5735 pg/ml [25–75% percentile, 4935–6302 (pg/ml)] was significantly higher than in the non-TPE group [2273 (pg/ml), 25–75% percentile, 1121–3832 (pg/ml), p ≤ 0.0001] (Table 3, Fig. 2D). This was associated with a high AUC value of 0.9342 (95% CI 0.8854–0.9830, p ≤ 0.0001) (Fig. 3). An optimal IL-6 cut-off value of 3260 (pg/ml) for PE was calculated using the ROC curve which gave a sensitivity of 100% (95% CI 88.43–100.0%) and a specificity of 67.24 (95% CI 53.66–78.99%) and a diagnostic accuracy for TPE of 78.4% (69/88) (Table 4).

The median CXCL8 concentration in the TPE group was 1038 (pg/ml) [25–75% percentile, 344.1–1853 (pg/ml)], which was significantly higher than with the non-TPEs [123 (pg/ml), 25–75% percentile, 67.75–485.6 (pg/ml), p ≤ 0.0001] (Table 3, Fig. 2E). This was associated with a moderate AUC value of 0.7905 (95% CI 0.6942–0.8868, p ≤ 0.0001) (Fig. 3). The optimal CXCL8 cut-off value of 144.5 (pg/ml) gave a sensitivity of 93.3% (95% CI 77.93–99.18%), a specificity of 58.6% (95% CI 44.93–71.40%) and a diagnostic accuracy of 70.45% (62/88) for differentiating of TPE from non-TPEs (Table 4). The median CCL1 levels in the TPE group were 565 (pg/ml) [25–75% percentile, 104.8–2353 (pg/ml)], which was significantly higher than in the non-TPE group [32.2 (pg/ml), 25–75% percentile, 12.2–61.98 (pg/ml), p ≤ 0.0001] (Table 3, Fig. 2F). This was associated with an AUC value of 0.905 [95% CI 0.8441–0.9662, p* ≤ *0.0001) (Fig. 3]. The optimal CCL1 cut-off value was 54 pg/ml which resulted in a sensitivity of 100% (95% CI 65.28–94.36%), a specificity of 70.69% (95% CI 34.95–61.78%) and a diagnostic accuracy for diagnosing TPE from non-TPEs of 80.68% (71/88) (Table 4).

The median IP-10 level in the TPE group was 2833 (pg/ml) [25–75% percentile, 909–4184 (pg/ml)], was significantly higher than in the non-TPE group [944.4 pg/ml, 25–75% percentile, 355.5–1970 (pg/ml), p = 0.0001] (Table 3, Fig. 2G). This was associated with a moderate AUC of 0.746 (95% CI 0.6350–0.8569, p = 0.0002) (Fig. 3). The optimal IP-10 cut-off value of 891.9 pg/ml for TPE resulted in a sensitivity of 83.33% (95% CI 65.28–94.36%), a specificity of 48.28% (95% CI 34.95–61.78%) and a diagnostic accuracy of 60.2% (53/88) (Table 4). Finally, the median IL-18 concentration in the TPE group was 2196 (pg/ml) [25–75%, 513.4–3035 (pg/ml)] was not significantly different from non-TPE groups [1003 pg/ml, 25–75% percentile, 577.8–2291(pg/ml), p = 0.1797] (Table 3, Fig. 2H). This was associated with a low AUC value of 0.5879 (95% CI 0.454–0.7219, p = 0.178) (Fig. 3). The optimal IL-18 cut-off value of 2285 (pg/ml) for differentiating TPE from non-TPE gave a sensitivity of 50% (95% CI 31.3–68.7%), a specificity of 75.9% (95% CI 62.83–86.13%) and a diagnostic accuracy of 67.04% (59/88) (Table 4).

Full details regarding the cut-off values, sensitivity, specificity, PLR, NLR, PPV, NPV and diagnostic accuracy of the above biomarkers are provided in Supplementary Table S1 and Supplementary Fig. S1 online.

### Combined ADA and IL-27 as a predictor of TPE versus non-TPE

Since IL-27 and ADA were the best individual predictors of TPE we determined whether combined IL-27.ADA would provide even greater predictive ability. The median ADA.IL-27 level in the TPE group was 208.5 × (10^3^ U ng/l^2^) [25–75% percentile, 178.2–261.6 × (10^3^ U ng/l^2^)], was significantly higher than in the non-TPE group [19.23 × (10^3^ U ng/l^2^) , 25–75% percentile, 13.3–25.42 × (10^3^ U ng/l^2^), p ≤ 0.0001] (Table 3, Fig. 2C). This was associated with a high AUC of 0.9994 (95% CI 0.9975–1.001, p* ≤ *0.0001) (Fig. 3). The ROC curve (Fig. 3) showed that ADA.IL-27 separated TPE from the non-TPE group better than either ADA (0.9471) or IL-27 (0.9879) alone. Furthermore, we determined the optimal ADA.IL-27 cut-off value in the pleural fluid as 42.68 × (10^3^ U ng/l^2^) by ROC curve analysis. With this cut-off value a sensitivity of 100% (95% CI 88.43–100.0%) a specificity of 98.28% (95% CI 90.76–99.96%) together with a PLR = 58, a NLR = 0.0, a PPV = 96.78 and a NPV = 100 was obtained for TPE diagnosis compared with non-TPEs. Thus, the diagnostic accuracy of IL-27.ADA levels in pleural effusion was 98.86% (87/88) (Table 4).

## Discussion

The current study demonstrated that the levels of ADA, CCL1, CXCL8, IL-6, IL-27 and IP-10 in pleural fluid were significantly higher in TPE compared to non-TPE samples. IL-18 levels did not differentiate between TPE and non-TPE subjects. The specific cut-off levels of ADA and IL-27 were identified and these had good sensitivity and specificity for TPE against non-TPE subjects. The combination of ADA.IL-27 improved the sensitivity, specificity and diagnostic accuracy compared to IL-27 and ADA alone in the diagnosis of TPE.

Macrophages identify microbial antigens via pattern recognition receptors and produce cytokines including IL-27. They thereby form a bridge between the innate and acquired immunity. IL-27 stimulates adaptive immune responses in lymphocytes and it is able for inducing CD4+ clonal proliferation in naïve CD4+ T cells. This cytokine, along with IL-12, causes the production of interferon-gamma in naïve CD4+ T cells^14,15^. TB enhances the production and secretion of IL-27 from antigen presenting cells (APCs) increasing its local concentration^16^. Our results confirm previous studies that have shown the high diagnostic value of IL-27 for the diagnosis of TPE^2,13,17,18^ with higher levels of IL-27 in TPE than in non-TPE patients^13,19^. Wu and colleagues reported that the diagnostic accuracy of IL-27 better than that seen with IFN-γ or with ADA showing a sensitivity of 95% and specificity of 97.6%^13^. A meta-analysis of IL-27 studies in TPE gave a pooled sensitivity of 0.92 and a specificity of 0.90^19^. In the current study, we found an equally high sensitivity and specificity of IL-27 for the diagnosis of TPE with a sensitivity and specificity significantly higher than that of the other biomarkers. In the study of Valdes and colleagues^18^, the sensitivity and specificity of IL-27 were lower than that of ADA. This contrast with the current study where the sensitivity and specificity of IL-27 was higher than that of ADA.

In a systematic review, Aggarwal and colleagues examined 174 publications with 27,009 patients. Importantly, all studies had a high risk of bias but suggested good sensitivity (0.92), specificity (0.9) and diagnostic odds ratio (97.42). Many studies (65) used an ADA threshold of 40 ± 4 (IU/l) which gave a good sensitivity (0.93) and specificity (0.90) whilst 4 studies using an ADA threshold of > 65 (IU/l) gave a sensitivity and specificity of 0.86 and 0.94, respectively^20^. An earlier meta-analysis indicated that the summary measures derived from ROC curves was 92.2% for both sensitivity and specificity^21^. In our study, with an ADA cut-off value > 27.5 (IU/l) gave a sensitivity of 90%, a specificity of 96.5% and a diagnostic accuracy 94.3%. The increase in ADA in TPE probably reflects macrophage activation downstream of greater CD4+ lymphocyte activation in pleural fluid^22^.

Previous reports have suggested that concurrent measurement of analytes can improve the diagnostic efficiency^23^. In a previous study, Valdes et al. reported that with an IL-27 cutoff point of 0.55 (ng/ml), there was a sensitivity of 91.4% and a specificity of 85.1%, which was significantly less than that for ADA, ADA-2, ADA.IL-27 or ADA-2.IL-27. However, the addition of IL-27 improved the sensitivity of ADA and ADA-2 to produce 100% sensitivity for both^18^. In the present study, the diagnostic efficiency of ADA and IL-27 individually for TPE was 94.3% and 97.72%, respectively and the combination of ADA and IL-27 (ADA.IL-27) resulted in a sensitivity of 100%. This makes the combined test potentially useful for the establishment of a clinical test for TPE diagnosis. The PLR and NLR are more clinically valid and in this study, the PLR was 58.13 and NLR was 0.0. This indicates that compared to patients without TPE, patients with tuberculous pleurisy have a 58-fold higher chance of being ADA.IL-27 assay positive. Similarly, if a patient gains a negative result of ADA.IL-27 assay, he could have a 0% chance of being a tuberculous pleurisy patient. The diagnostic efficiency was improved 4.56% (98.86%) when ADA and IL-27 were used together.

Other analytes that we measured were also significantly different between TPE and non-TPE subjects. For example, CCL1 or I-309 is a monocyte attractant^24^ and has been implicated in the formation and maintenance of granuloma following Mtb infection^25^. CCL1 levels were significantly raised in the plasma of patients with pulmonary tuberculosis^26^. The current study suggests that CCL1 may predict TPE compared to non-TPE subjects with 100% sensitivity and a specificity of 70.7%. Our data suggests that compared to patients without TPE, patients with tuberculous pleurisy have a threefold higher chance of being CCL1 assay positive. Similarly, if a patient gains a negative result of CCL1 assay, he could have a 0% chance of being a tuberculous pleurisy patient. However, its diagnostic value (80.68%) was much less than that of ADA, IL-27 and combined ADA.IL-27.

There were some limitations to this study including the restricted number of patient samples and the analysis of only four PE disease groups. In addition, it would have been ideal to have used pre-defined thresholds in our analysis. However, our study indicates an excellent predictive value for individual measures of IL-27 (97.72%) and ADA (94.3%) with a high sensitivity and specificity for the differential diagnosis of TPE. In addition, we show an even greater accuracy of ADA.IL-27 as a predictive marker of TPE (98.86%). Our data demonstrate that the measurement of soluble mediators obtained from pleural fluid samples may provide high levels of sensitivity and specificity for pleural diseases that may be applicable for the development of a rapid and non-invasive diagnostic test. The specific thresholds defined here should be used in a subsequent cohort to test their utility.

In summary, we have demonstrated elevation of IL-27, ADA and several other cytokines and chemokines in TPE pleural fluid which reflects immune activation in response to *M*. *tuberculosis* infection. Our data suggests that the combination of IL-27 and ADA is more valuable in the diagnosis of TPE compared to non-TPE than either is alone. The measurement of this combination of soluble mediators in pleural fluid that provide high levels of sensitivity and specificity for TPE may be applicable for the development of a rapid and non-invasive diagnostic test that may prove to have good clinical utility. Future studies are needed to confirm the potential of this combined biomarker for the rapid detection of TPE.

## Materials and methods

### Patient selection

The study protocol was approved by the Institutional Review Board for human studies of clinic center from Masih Daneshvari Hospital, Tehran, Iran; and informed written consent was obtained from all subjects or their legal guardians. The study was carried out in accordance with the approved Ethics (Ethic code: IR.SBMU.MSP.REC.1397.584). All experiments were performed in accordance with relevant guidelines and regulations. From December 2018-December 2019, 190 consecutive patients with PEs of unknown causes were enrolled. After a clinical and laboratory diagnosis, we excluded patients with transudates, samples of unknown origin, patients with more than one possible etiology of effusion and PEs with miscellaneous etiology from the study. Exudates were characterized using the Light’s criteria^27^. Patients were referred to the Infection wards of the Massih Daneshvari Hospital from across Iran.

Inclusion criteria included: (a) no invasive procedures to the pleural cavity, (b) not receiving anti-tuberculosis therapy, and (c) not suffering from lung trauma for three months prior to hospitalization. At the time of sampling, none of the patients received antibiotic therapy, anti-tuberculosis drugs, anti-malignancy treatments, corticosteroids, or non-steroidal anti-inflammatory drugs.

PEs were classified into TPE, MPE, EMP and PPE. TPEs fulfilled one or more of the following: (a) positive pleural fluid or pleural biopsy or sputum Ziehl–Neelsen stain or Lowenstein–Jensen culture, (b) caseous necrotic granulomas on pleural biopsy. MPEs were diagnosed by the discovery of malignant cells on pleural fluid cytology or pleural biopsy. No subjects with pleural mesothelioma or lymphoma were included in the study. Characterization of PEs from malignant patients was performed based on light microscopy. In addition, no samples from patients with a combination of MPE and TPE were included in the study.

EMPs were diagnosed by the presence of frank pus in their pleural effusions or smear or positive bacterial or fungal culture of pleural fluid (except for *Mtb*). The diagnosis of PPEs was based on negative pleural fluid bacterial culture, pH < 7.2 and pleural fluid glucose < 600 (mg/dl).

### Sample collection and processing

Pleural fluid (5 ml) was collected in heparin-containing tubes by thoracocentesis within 24 h of hospitalization and immediately placed in ice. Tubes were centrifuged at 1200×*g* for 5 min and mononuclear cells isolated by Ficoll-Hypaque gradient (Pharmacia, Uppsala, Sweden) within 1 h. Total and differential cell counts, protein, lactate dehydrogenase (LDH), ADA, glucose, cytology, and bacterial examination were evaluated in the biochemistry laboratory of the Masih Daneshvari Hospital. In addition; the cell-free supernatants of pleural fluid were frozen at − 80 °C immediately after centrifuge for later determining concentrations of cytokines by ELISA.

### Measurement of ADA, cytokines and chemokines

The amino radical of adenosine hydrolysed by ADA produces inosine and ammonia. When a-ketoglutaric acid and NADPH are added to the ammonia, l-glutamine and NADP+ are produced due to the reaction of glutaminic-acid dehydrogenase, which reduces the NADPH. This was determined by measuring the reduction in light absorption at 340 nm to evaluate ADA^7^. The concentrations of IL-6 and IL-27 were measured by enzyme linked immunosorbent assay (ELISA) (R&D SYSTEM, Minneapolis, MN, US). The concentrations of IL-18, CCL1, and IP-10 were measured by ELISA (Invitrogen by Thermo Fisher Scientific, Vienna, Austria). CXCL8 was measured by ELISA (BD Biosciences, CA, USA) according to the manufacturer’s protocol.

### Statistical analysis

Analysis was performed using SPSS version 16.0 (SPSS, Inc. Chicago, USA) and GraphPad Prism software (version 6; 07 GraphPad Software, Inc.). Non-parametric Mann–Whitney U test (median, 95% confidence intervals (CI) was used for the non-normally distributed variables and a t test (mean ± SEM) used for normally distributed variables. Receiver operating characteristic (ROC) curve analyses were used to evaluate the capacity of ADA and other biomarkers to differentiate TPE from non-TPE. The area under the ROC curve (AUC) was calculated and 95% confidence intervals (CIs) were used to test whether the hypothesis that the analyte could distinguish TPE from non-TPE (AUC > 0.5). ROC analysis also identified the optimal cut-off value for each analyte. P values < 0.05 were considered statistically significant.

## Supplementary Information

Supplementary Information
