# Supplementary material for: Potential diagnostic value of pleural fluid cytokines levels for tuberculous pleural effusion
Source: Sci Rep. 2021 Jan 12;11:660. doi: 10.1038/s41598-020-79685-1 (PMC7803752; doi:10.1038/s41598-020-79685-1)
Supplement: Supplementary file 1 — Supplementary Information [file 41598_2020_79685_MOESM1_ESM.pdf]

Potential diagnostic value of pleural fluid cytokines levels for tuberculous pleural effusion

Neda Dalil Roofchayee[1], Majid Marjani[2], Neda K.Dezfuli[1,5], Payam Tabarsi[2], Afshin Moniri [2], Mohammad Varahram [3], Ian M. Adcock[4], Esmaeil Mortaz[1, 2]

[1]Department of Immunology, Faculty of Medicine, Shahid Beheshti University of Medical Sciences, Tehran, Iran; [2]Clinical Tuberculosis and Epidemiology Research Center, National Research Institute of Tuberculosis and Lung Diseases, Shahid Beheshti University of Medical Sciences, Tehran, Iran; [3]Mycobacteriology Research Center, National Research Institute of Tuberculosis and Lung Diseases (NRITLD), Shahid Beheshti University of Medical Sciences, Tehran, Iran; [4]Respiratory Section, National Heart and Lung Institute, Faculty of Medicine, Imperial College London, London, United Kingdom, Priority Research Centre for Asthma and Respiratory Disease, Hunter Medical Research Institute, University of Newcastle, Newcastle, New South Wales, Australia; [5]Department of Immunology, School of Medicine, Dezful University of Medical Sciences, Dezful, Iran

Corresponding author: Esmaeil Mortaz

Email address: emortaz@gmail.com

Supplementary table S1. The diagnostic accuracy of pleural fluid inflammatory mediators for the differentiation of tuberculous from malignant pleural effusions.

Variables	Cut-off value	Area under curve (95% confidence interval)	P value	Sensitivity (%)	Specificity (%)	Positive likelihood ratio	Negative likelihood ratio	Positive predictive value	Negative predictive value	Diagnostic accuracy (%)
**IL-27**	1040 pg/ml	1	≤0.0001	100	100	-	0	100	100	100
**IL-6**	3026 pg/ml	0.99	≤0.0001	100	90	10	0	90.9	100	95
**IL-18**	1200 pg/ml	0.7778	0.0002	60	100	-	0.4	100	71.4	80
**CXCL-8**	144.5 pg/ml	0.7832	0.0002	93.3	60	2.33	0.11	70	90	76.6
**CCL-1**	51.85 pg/ml	0.9867	≤0.0001	100	93.3	14.28	0	93.7	100	96.6
**IP-10**	2072 pg/ml	0.8244	≤0.0001	60	100	-	0.4	100	71.4	80
**ADA**	20.5 IU/L	1	≤0.0001	100	100	-	0	100	100	100
**ADA.IL-27**	31.14 10^3.U.ng/L^2	1	≤0.0001	100	100	-	0	100	100	100

Abbreviations: ADA: Adenosine deaminase, IL: Interleukin, CXCL: C-X-C motif chemokine ligand, CCL: C-C motif chemokine ligand, IP-10: Interferon gamma-induced protein 10 or CXCL10.

Supplementary figure S1:

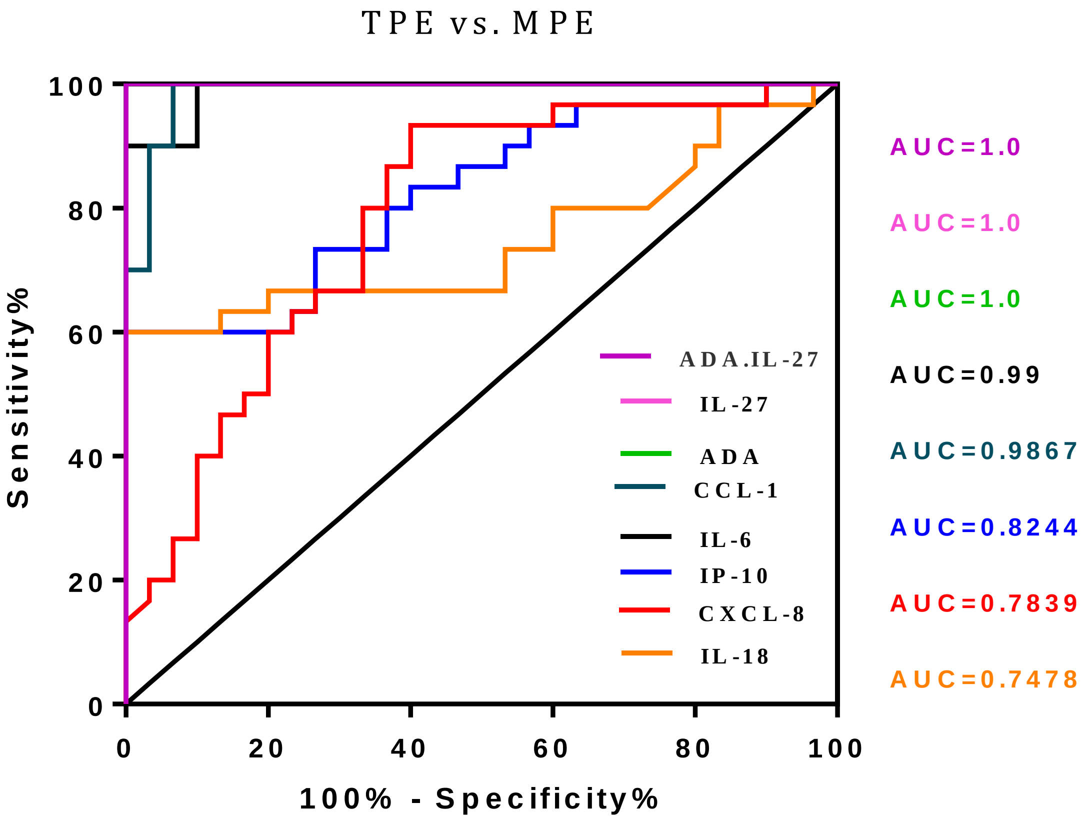

Supplementary Figure S1:

ROC curve of ADA, IL-27, ADA-IL-27, IL-6, IL-18, CXCL8, CCL1 and IP-10 for differential diagnosis of TPE (n = 30) versus MPE (n = 30). TPE: Tuberculous pleural effusion. MPE: Malignant pleural effusion.
